# HCMV IE1/IE1mut Therapeutic Vaccine Induces Tumor Regression via Intratumoral Tertiary Lymphoid Structure Formation and Peripheral Immunity Activation in Glioblastoma Multiforme

**DOI:** 10.1007/s12035-024-03937-8

**Published:** 2024-01-23

**Authors:** Xiaoli Yang, Shasha Jiang, Fengjun Liu, Zonghui Li, Wenxuan Liu, Xianjuan Zhang, Fulong Nan, Jun Li, Meng Yu, Yunyang Wang, Bin Wang

**Affiliations:** 1 Department of Pathogenic Biology, School of Basic Medicine, Qingdao University, Qingdao, China; 2 Department of Clinical Laboratory, The Affiliated Hospital of Qingdao University, Qingdao, People’s Republic of China; 3 Department of Endocrinology and Metabolism, Affiliated Hospital of Qingdao University, Qingdao, China

**Keywords:** GBM, HCMV IE1/IE1mut vaccine, Immunity therapy, TLS, CTL

## Abstract

Glioblastoma multiforme (GBM), a highly malignant invasive brain tumor, is associated with poor prognosis and survival and lacks an effective cure. High expression of the human cytomegalovirus (HCMV) immediate early protein 1 (IE1) in GBM tissues is strongly associated with their malignant progression, presenting a novel target for therapeutic strategies. Here, the bioluminescence imaging technology revealed remarkable tumor shrinkage and improved survival rates in a mouse glioma model treated with HCMV IE1/IE1mut vaccine. In addition, immunofluorescence data demonstrated that the treated group exhibited significantly more and larger tertiary lymphoid structures (TLSs) than the untreated group. The presence of TLS was associated with enhanced T cell infiltration, and a large number of proliferating T cells were found in the treated group. Furthermore, the flow cytometry results showed that in the treatment group, cytotoxic T lymphocytes exhibited partial polarization toward effector memory T cells and were activated to play a lethal role in the peripheral immunological organs. Furthermore, a substantial proportion of B cells in the draining lymph nodes expressed CD40 and CD86. Surprisingly, quantitative polymerase chain reaction indicated that a high expression of cytokines, including chemokines in brain tumors and immune tissues, induced the differentiation, development, and chemokine migration of immune cells in the treated group. Our study data demonstrate that IE1 or IE1mut vaccination has a favorable effect in glioma mice models. This study holds substantial implications for identifying new and effective therapeutic targets within GBM.

**Supplementary Information:**

The online version contains supplementary material available at 10.1007/s12035-024-03937-8.

## Introduction

In adults, glioblastomas, which are the most common primary brain tumors, remain uniformly lethal with a median survival of 12 months [1]. However, despite the availability of well-established approaches, the clinical benefits have been rather limited, and the life expectancy of GBM patients has only seen a marginal extension to approximately 15 months. The current standard of care for glioma includes maximal resection followed by concurrent radiation and temozolomide (TMZ) chemotherapy within 30 days of surgery [2], radiotherapy, and corticosteroid treatment; but all of these treatments have immunosuppressive effects. Novel immunotherapy strategies have shown considerable effectiveness in clinical trials, but some flaws were also exposed during the treatment process. For instance, chimeric antigen receptor T cells (CAR-T), the most promising treatment, but evidence from a clinical trial on many solid tumors revealed that CAR T alone have insufficient antitumor activity [3]. Vaccination has been considered one of the most promising approaches to improving the outcomes of patients with glioblastoma [4]. In comparison to whole proteins and tumor lysates, recombinant proteins can be synthesized relatively easily in large quantities at GMP grade and has low risks for antigen-induced anaphylaxis, and they are particularly suited for developing personalized tumor vaccines [5].

HCMV is one of the β-herpesviruses that cause severe illness and even death in organ transplant patients or immunocompromised people [6]. Antiviral therapy and immunotherapy against HCMV have been shown to enhance the prognosis of GBM patients in clinical trials [7]. Moreover, HCMV IE1 has been identified in specimens obtained from glioma patients but not in brain areas of normal individuals [8], and numerous studies have revealed a strong correlation between glioma and HCMV IE1 [9, 10]. Hence, HCMV IE1 has been proposed as a promising therapeutic target. Nevertheless, Soroceanu et al. [9] found that IE1 can promote the malignant progression of glioma. Thus, to ensure vaccine safety, we further developed the IE1 mutated vaccine (IE1mut) to reduce its potential harmful effects on humans.

Tertiary lymphoid structures (TLSs), or ectopic lymphoid structures, are organized aggregates of immune cells and are formed in non-lymphoid tissues post-birth [11]. TLSs do not naturally occur under physiological conditions but are formed in a chronic inflammatory environment. Although the specific composition of TLSs varies, they share structural and functional characteristics with secondary lymphoid structures, such as lymph nodes, and contain B-cell follicles and germinal centers surrounded by a T-cell region [12]. Furthermore, various immune cell infiltrates associated with the development of adaptive immune responses are observed in regions involved in diverse chronic inflammatory conditions, including B-cell areas containing dendritic cells (DCs), follicular dendritic cell germinal centers, and proliferating T cells. The same set of molecules, including lymphotoxin (LT)-α and LT-β [13], as well as the lymphoid chemokines Ccl19 and Ccl21, chemokine CXC ligand 13 (Cxcl13), and tumor necrosis factor-alpha (TNF-α), play an essential role in TLS and lymph node formation [14]. In the context of cancer, TLS serves as an alternative site for lymphatic drainage and a site for antigen presentation and initial T-cell activation. Notably, the presence of TLS has been reported to be associated with a favorable prognosis and extended survival in solid tumors, such as lung, melanoma, prostate, bladder, and breast cancers [15]. However, TLS in gliomas has been rarely described so far. And GBM is a notoriously immunosuppressive (“cold”) tumor with T-cell exhaustion [16], as its number of CD8^+^T cells is exceptionally low and the remaining T cells exhibit an unresponsive status to antigen challenge [17]; reliving the immunosuppressive microenvironment is a great challenge for GBM.

In the present study, we successfully established a murine glioma model expressing HCMV IE1 and developed therapeutic glioma vaccine IE1and IE1mut. The results revealed that therapeutic vaccination with IE1 or IE1mut efficiently mobilized and stimulated immune cells in the peripheral immune organs and central nerves, thereby promoting the formation of TLS within the gliomas, in which IE1mut reduces IE1’s potential toxicity. Consequently, tumor regression was observed, which led to a considerable survival prolongation in the murine glioma model. Overall, our research provides a new and effective method for treating gliomas that holds promise for clinical applications, offering an additional treatment for glioma patients.

## Materials and Methods

### Animals

Six- to ten-week-old female C57BL/6J mice were purchased from SPF (Beijing) Biotechnology Co., Ltd. and raised in the SPF animal room of Qingdao University’s Medical Department. All mice were housed and maintained under specific pathogen-free conditions at 23°C ± 2 °C on a 12-h day/night cycle and received an autoclaved standard diet and water ad libitum. All experimental procedures involving the mice followed the National Institutes of Health’s standards for the care and use of experimental animals. The study received approval from Qingdao University’s Animal Experiments Committee.

### Cell Culture and Construction of IE1 Overexpression Cell Line

The GL261and GL261-Luc were purchased from the Shanghai icellbioscience Biotechnology Corporation. Both cell lines were cultured in Dulbecco’s Modified Eagle Medium (DMEM) supplemented with 10% fetal bovine serum (Gibco) and 1% penicillin-streptomycin at 37°C in a humidified incubator with 5% CO_2_. For overexpression of the human cytomegalovirus IE1 protein, a lentiviral vector was synthesized and designed by Shanghai GeneChem Co., Ltd, and the fragment containing the full-length of IE1 was introduced into the lentiviral vector by molecular biology. The specific primers used for IE1 amplification were Forward Primer: GTTGGCCGAAGAATCCCTCA; Reverse primer: TATGCCGCACCATGTCCACT. The vector enables the overexpression of the *IE1* gene, direct transfection of GL261-Luc cells, and subsequent screening of positive cells using puromycin, resulting in the establishment of stable glioma cell lines with enhanced expression of IE1.

### Protein Synthesis

IE1 and IE1-mut recombinant proteins were synthesized by GenScript Biotech Corporation, following their respective amino acid sequences. The expression of both recombinant proteins was achieved using a prokaryotic expression system, and subsequent purification was conducted using nickel columns, resulting in protein purity >75% as confirmed by SDS-PAGE analysis. Then, the recombinant proteins were dissolved in 50 mM Tris-HCl, 500 mM NaCl, 10 % Glycerol, and pH 8.0 and stored at −80 °C for long-term preservation.

### Mouse Immunization

To assess the *in vivo* safety of IE1 and IE1mut, female wild-type C57BL/6J mice (6–8 weeks old) were obtained from SPF (Beijing) Biotechnology Co. Ltd. Then, they were randomly separated into three groups (*n* = 6 per group): PBS, IE1+AddaVax, IE1-mut+AddaVax groups. The test group received intramuscularly vaccine with 30 μg/mouse of each vaccine antigen. After the first immunization on day 0, the same amount of recombinant proteins were administered on days 14 and 28 [18]. On day 35, the mice were euthanized, and blood and other organs were collected for further investigations.

### Orthotopic Murine Glioma Models

Female C57BL/6J mice (SPF), aged 6 to 10 weeks, were used in our orthotopic glioma experiments as previously described [19]. To establish the syngeneic gliomas, we removed the hair of the anesthetized mouse’s head with an animal shaver, disinfected the scalp with iodophor, and fixed the mice on a mouse stereotaxic injector (RWD Life Science, CA). Then, the scalp was cut open, and the surface tissue was removed with medical H_2_O_2_ to expose the skull surface. The holes were drilled on the skull surface with a hand-held cranial drill according to the positioning described in previous study [20] (0.5 mm anterior, 2.1 mm lateral from bregma, and 3.2 mm deep from the bregma). Furthermore, using a micro-pump injector and a 5-μl Hamilton syringe equipped with a 33-gauge needle, 1 × 10^5^ GL261-Luc-IE1 cells (2μL phosphate-buffered saline [PBS], Beijing Solarbio Science & Technology Co., Ltd.) were stereotactically injected into the right striatum for 4 min at a rate of 0.5 μL/min. Following implantation, cells were allowed to settle for 5 min, and the needle was slowly withdrawn from the skull. The hole was then sealed with a sterile bone wax. On day 5 after the implantation, tumor burden was monitored via luciferase imaging. Subsequently, the mice were randomly assigned to the treatment groups (IE1+AddaVax and IE1mut+AddaVax groups) based on tumor radiance, ensuring that the average tumor radiance in each group was approximately equivalent. When the animals showed predetermined features of neurologic deficits (failure to ambulate, weight loss >20% body mass, lethargy, hunched posture), they were sacrificed. In survival experiments, each group had six to eight mice. All experiments were repeated at least in thrice. At the experimental end-point (days 20–25), mice were killed by anesthetizing for cardiac perfusion with 30 mL PBS and 20 mL 4% paraformaldehyde (PFA, Wuhan Servicebio Technology Co., Ltd). At the survival end-point, the mice were sacrificed by cervical dislocation.

### *In Vivo* Vaccine Therapies

After the glioma mice were observed for tumor growth via luciferase imaging at 5 days after implantation, they were randomly divided into different treatment groups, and the average tumor radiance in each group was roughly equivalent. On the 7th day after tumor-bearing, the upper limb muscles of mice in the different experimental groups were injected with different proteins plus adjuvant, with a final dose of 100 μl/mice. In contrast, mice in the control group were injected with the same volume of sterile PBS at the same volume site. Both the protein and adjuvant were diluted in sterile PBS. The mice received repeated doses on days 14 and 28 after tumor implantation.

### Bioluminescence Imaging

For bioluminescence imaging (BLI) studies, luciferase activity was monitored using an IVIS Lumina II instrument (Perkin Elmer, USA). On 5 days, post-implantation of GL261-Luc cells in the brain, mice were anesthetized and injected i.p. with 200 μL (Add the corresponding volume of 15 mg/ml fluorescein working solution at a concentration of 10μl/g body weight) of the bioluminescent substrate D-luciferin, sodium salt (Yeasen Biotechnology Co., Ltd.). Images were obtained at 10–20 min after i.p. injection of D-luciferin. To photograph mice, the following criteria were used: medium binning and F/Stop:1, field of view D, and exposure time 60 s. Results of *in vivo* imaging were analyzed using Living Image Software (Perkin Elmer).

### Quantitative Real-Time PCR

Total brain tumor or immune organs’ RNA was extracted by TRIzol reagent (Invitrogen, Carlsbad, CA) following the manufacturer’s instructions. The quantity and purity of RNA were analyzed using Bioanalyzer 2100 and the RNA 6000 Nano LabChip Kit (Agilent, Folsom, CA). The cDNA was reverse transcribed with a reverse transcription kit (Nanjing Vazyme Biotech Co., Ltd.R212) according to the manufacturer’s instructions. and SYBR Green Fast qPCR Mix (Vazyme, Q321) was added for qPCR amplification. All primer sequences are shown in Table 1 Quantitative real-time experiments were performed in three independent experimental replicates, and the comparative Ct (2^^-ΔΔCt^) method was used to measure the relative amount of target mRNA.

**Table 1:** The sequence of the primers for quantitative real-time PCR

Gene	Forward primer, 5’-3’	Reverse primer, 5’-3’	Condition
*GAPDH*	CCAGCAAGGACACTGAGCAA	GCCCCTCCTGTTATTATGGGG	Stage1:95°C, 30 s. Stage2:40 cycles of 95°C for 10 s, 60°C for 30 s. Stage3:95°Cfor 15 s, 60°C for 60 s, 95°C for 15s.
*Cxcl13*	GGCCACGGTATTCTGGAAGC	GGGCGTAACTTGAATCCGATCTA	
*Ccl19*	ACCACACTAAGGGGCTATCAG	TTCTTCAGTCTTCGGATGATGC	
*Ccl21*	GCTGCAAGAGAACTGAACAGACA	CGTGAACCACCCAGCTTGA	
*Lta*	GCATCTTCTAAGCCCTGGGGG	TGTCATGTGGAGGACCTGCTGTG	
*Ltb*	GTTCAACAGCTGCCAAAGGG	CATCCAAGCGCCTATGAGGT	
*TNF-α*	CGCTCTTCTGTCTACTGAACTTCGG	GTGGTTTGTGAGTGTGAGGGTCTG	
*IFN-γ*	ATGAACGCTACACACTGCATC	CCATCCTTTTGCCAGTTCCTC	
*IL-12p40*	CATTGAACTGGCGTTGGAAGCAC	GGGCGGGTCTGGTTTGATGATG	

### SDS-PAGE Analysis

The IE1 or IE1mut recombinant protein was thoroughly mixed with 5× loading buffer to ensure even distribution, followed by boiling in water for 10 min to denature the protein. Then 10% SDS-PAGE gel was prepared, and 10 μg of the denatured protein was loaded into each well. Electrophoresis was then performed, and after electrophoresis, IE1 and IE1mut proteins were detected using Coomassie brilliant blue staining.

### Isolation of Immune Cells from Spleen and Lymph Node

At 3–7 days after three immunizations, the mice were sacrificed via cervical dislocation, and their whole spleens and draining lymph nodes were aseptically collected. The tissues were extracted under aseptic conditions, the coating was torn off, and the tissues were cut into small pieces with ophthalmic scissors. A nylon screen or cell filter screen was placed on the plate, and a small amount of PBS was added to ensure that the organ tissues and obtained cells remained in a liquid environment. The organ tissue was placed on the screen, and sterile tweezers were used to grind the organ tissue. The grinding force should be controlled as much as possible, and the screen should be kept suspended to avoid mass cell death caused by direct grinding on the bottom of the dish. After grinding, PBS was used to wash the screen. The cell suspension was collected and filtered through a 70-μm sterile cell filter to obtain a single cell suspension (a red blood cell lysate buffer was used to remove the red blood cells from the spleen).

### Flow Cytometry and FACS

Intracellular cytokine staining analysis was performed as described previously [21]. Antibodies specific for cell surface staining were used. The following antibodies were used: Pacific Blue™ anti-mouse CD3 Antibody (BioLegend Cat. No. 100213), FITC anti-mouse CD4 Antibody (BioLegend Cat. No. 130308), Brilliant Violet 605 anti-mouse CD4 (Biolegend, cat.100451), APC/Cyanine7 anti-mouse CD8a Antibody (BioLegend Cat. No. 126620), PE/Cyanine7 anti-mouse CD69 Antibody (BioLegend Cat. No. 104512), APC anti-mouse CD107a (LAMP-1) Antibody (BioLegend Cat. No. 121614), FITC anti-mouse CD11c Antibody (BioLegend Cat. No. 117305), APC anti-mouse/human CD11b Antibody (BioLegend Cat. No. 101211), Pacific Blue™ anti-mouse CD86 Antibody (BioLegend Cat. No. 105021), FITC anti-mouse CD19 Antibody (BioLegend Cat. No. 101505), PE anti-mouse TNF-α Antibody (BioLegend Cat. No. 506305), and PE anti-mouse IFN-γ Antibody (BioLegend Cat. No. 505807). All data were acquired by Beckman CytoFLEX (Beckman Coulter, Brea, CA, USA) and analyzed with Flow Jo V10.

### H&E Staining

Brain tumor tissue samples were embedded in paraffin wax, cut into 3–5-μm tissue sections, stained with H&E according to the manufacturer’s instructions, and observed by a light microscope for brain tissue structure.

### Immunofluorescent Staining of Mouse Sample

After cardiac perfusion of anesthetized mice, the brains were collected and fixed overnight in 4% paraformaldehyde fixative (PFA) followed by gradient dehydration in 15% and 30% sucrose solutions, respectively. Brain tissues were embedded in paraffin and cut into 3–6-μm tissue sections. Paraffin sections were deparaffinized and hydrated through a series of xylene and ethanol gradient concentrations. They were then placed in water for 5 min, followed by antigen retrieval using a 0.01M pH6.0 citric acid repair solution for 10 min. Sections were blocked with a solution containing 5% bovine serum albumin (BSA) and 0.1% Triton X-100 for 30 min. Following blocking, the sections were incubated overnight at 4°C with the primary antibody of interest. The next day, the sections were washed with PBS, blocked for 15 min, and incubated with a secondary antibody at 1:500 for 60 min at room temperature. After washing, the sections were treated with an anti-fluorescence attenuation blocker containing DAPI for 10 min. Coverslips were added, and the images were observed using an Olympus Fluoview FV1200 microscope, and the image were analyzed with ImageJ software (National Institutes of Health, Bethesda, MD). The information on primary resistance is as follows: CD3 (1:50, cat. NB600-1441SS; NOVUS, Centennial, CO), CD4 (1:200, cat. NBP1-19371SS; NOVUS, Centennial, CO), CD8 (1:100, cat. NBP1-49045SS; NOVUS, Centennial, CO), Ki67 (1:100, ABclonal, cat. A16919, Wuhan, China), CD107a (1:500, cat. GB114929, Servicebio, Wuhan, China), TNF-α (1:300, cat. GB11188, Servicebio, Wuhan, China), CD11c (1:200, cat. GB11059, Servicebio, Wuhan, China), and F4/80(10 μg/mL, cat.14-4801-82, Thermo Fisher).

### Statistical Analysis

All statistical analysis was performed using GraphPad Prism 8.0 (GraphPad, La Jolla, CA, USA). Kaplan–Meier curves were analyzed by using the log-rank test. Data are represented as means ± SEM and were analyzed by one-way ANOVA. *p* < 0.05 was accepted as statistically significant.

## Results

### Preparation of IE1 and IE1mut Recombinant Protein

**Fig. 1:**
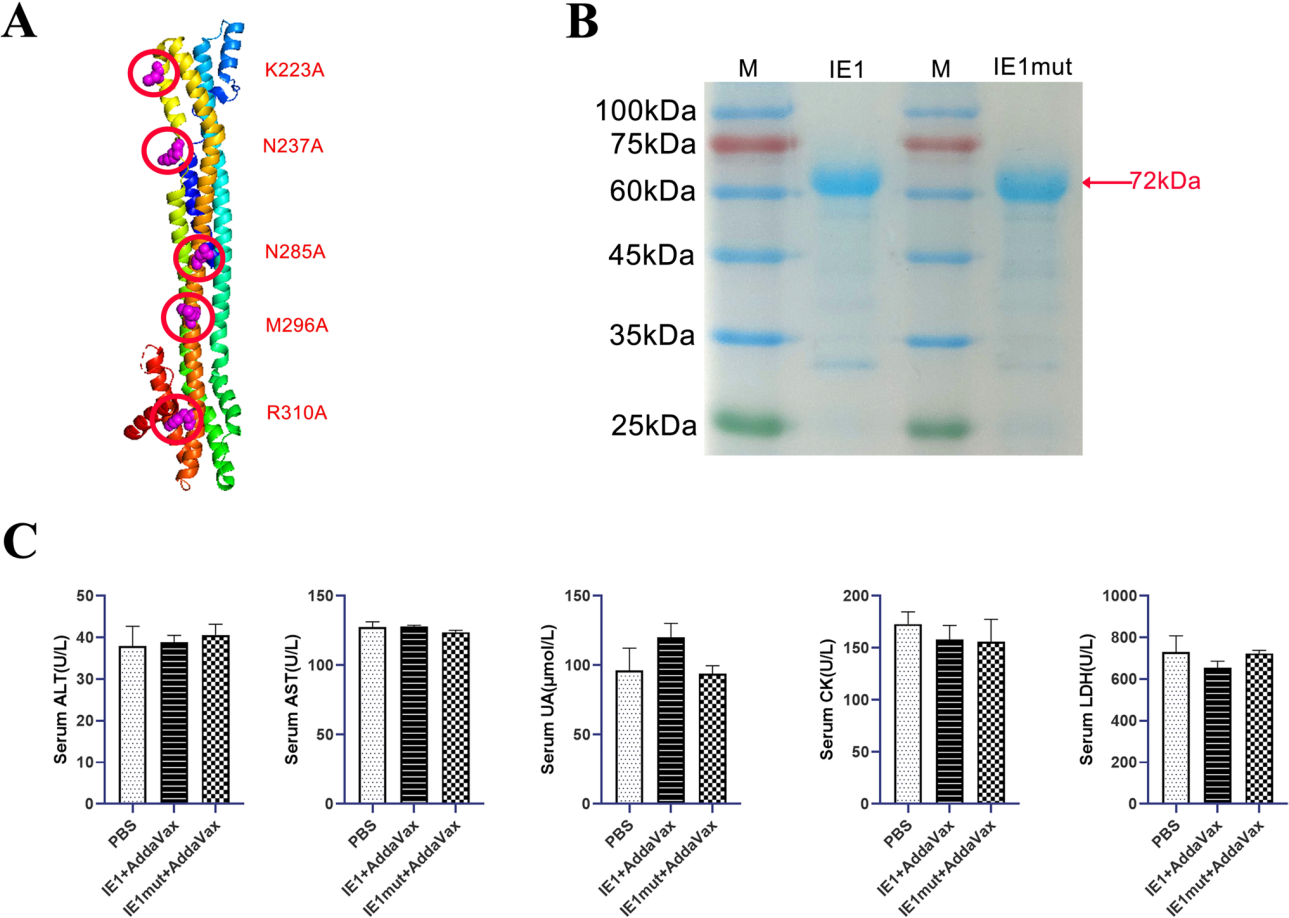
IE1 or IE1mut protein structure prediction and verification and toxicity detection to the body. **A** IE1 core structure diagram. Regions mutated to generate IE1mut are indicated in red. **B** IE1 and IE1mut protein were stained with Coomassie brilliant blue after SDS-PAGE. **C** Collect the serum of mice 7 days after three times of immunization to detect the biochemical indexes of mice including AST, ALT (liver function index), UA (renal function index), CK, LDH (heart function index). *n*=4–6 mice per group. There was no statistically significant difference between the groups. Bars: mean ± SEM. The one-way ANOVA was used to analyze statistical differences

HCMV IE1-72 kDa increased the flap endonuclease 1(FEN1) stability, which causes human fibroblasts that were infected to grow more slowly with low production. Contrarily, the FEN1 binding-deficient mutations K223A, N237A, N285A, M296A, and R310A of IE1mut were incapable of increasing the FEN1 stability [22]. And mutation of K450 to arginine (K450R) results in loss of IE1-72 kDa SUMOylation [23]. For investigating of the impact of IE1 and IE1mut on cells, we employed lentiviral vectors to infect the mouse and human glioma cell lines, thereby establishing cell lines with IE1 and IE1mut overexpression. CCK-8, cell cycle, and transwell data revealed that IE1 boosted glioma migration, proliferation, and viability in both human and mouse gliomas (Supplementary Fig. 1). Based on the sequence of IE1 protein, we used PyMOL to predict the 3D structure of the IE1mut protein after mutating the amino acid site (Fig. 1A). The IE1 and IE1mut recombinant proteins were purified via His-tag-based affinity purification. The molecular weight of these recombinant proteins was approximately 72 kDa (Fig. 1B).

To assess the safety of the developed IE1/IE1mut vaccines, mouse serum was collected at 7 days after three immunizations for biochemical analysis and potential toxicity evaluation in the heart, liver, and kidney. The biochemical parameters (AST, ALT, UA, CK, and LDH) of all groups were within the normal range, similar to those of the control (PBS) group, indicating that the protein had no obvious toxic and side effects on mice (Fig. 1C).

### IE1 or IE1mut Protein Therapy Induces Tumor Regression and Prolongs Survival

**Fig. 2:**
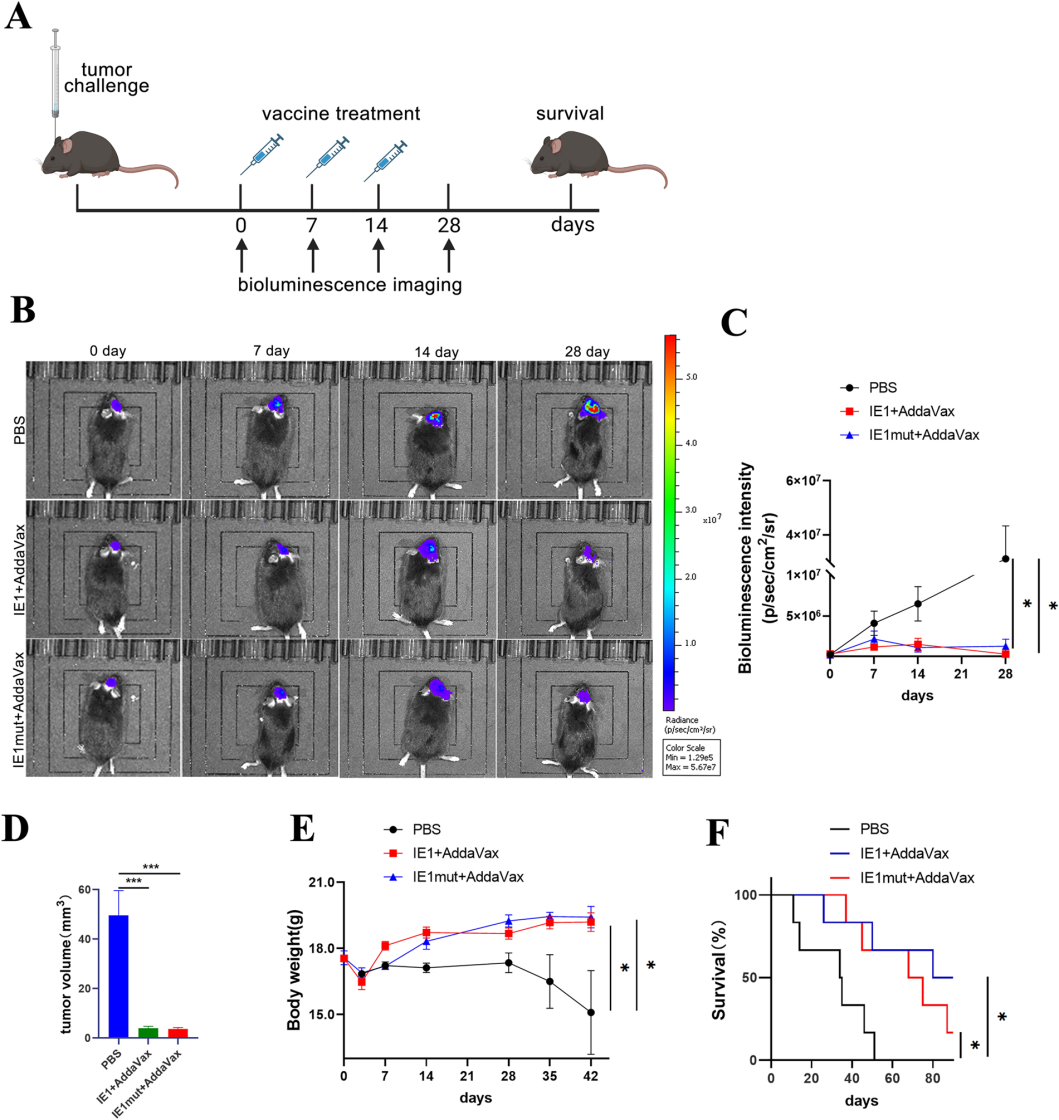
Observation of curative effect of IE1 or IE1mut. **A** Immunotherapy regimen and bioluminescence imaging time point of glioma model mice. **B** Bioluminescence imaging of representative brain tumors of tumor-bearing mice at various time points after representative immunotherapy. **C** Changes in the bioluminescent intensity of tumors in the experimental group and the control group at each time point. **D** The volume brain tumors were quantified in each group after vaccine treatment. **E** Evaluation of body weight changes over time in the experimental group and the control group after tumor-bearing. **F** Kaplan–Meier survival curve of GL261tumor-bearing mice treated with IE1 or IE1mut; log-rank test. *n*=4–6 mice per group. Bars: mean ± SEM. The one-way ANOVA was used to analyze statistical differences. *p < 0.05

To observe the therapeutic effect of IE1 or IE1 protein on tumor-bearing mice, we used a live imaging technology to observe the growth before protein injection and on the 7th day after each protein injection. Subsequently, treatment was initiated in tumor-bearing mice, and their survival was monitored over time. At 7 days after the initial dose of protein immunization, no notable difference in the tumor growth was observed between the treated and control groups. Interesting, on the 14th day, the tumor size of the tumor-bearing mice in the two treated groups was reduced compared with that in the control group. The difference became more pronounced by the 28th day, with the mice in the experimental group exhibiting significantly smaller tumor than those in the control group (Fig. 2B, C). Regarding brain tumor volume, the experimental group exhibited significantly reduced tumor infiltration as compared to the control group (Fig. 2D). Moreover, as the brain tumors proliferated, the mice began to lose weight before dying (Fig. 2E). Compared with control group, both IE1- and IE1mut-treated groups demonstrated an improvement in the survival rate following three doses of protein immunization (Fig. 2F). Overall, these findings suggest that IE1/IE1mut inhibits the growth of glioma tumors in glioma mouse models.

### IE1 or IE1mut Enhanced TLS Formation in Murine Glioma Models

**Fig. 3:**
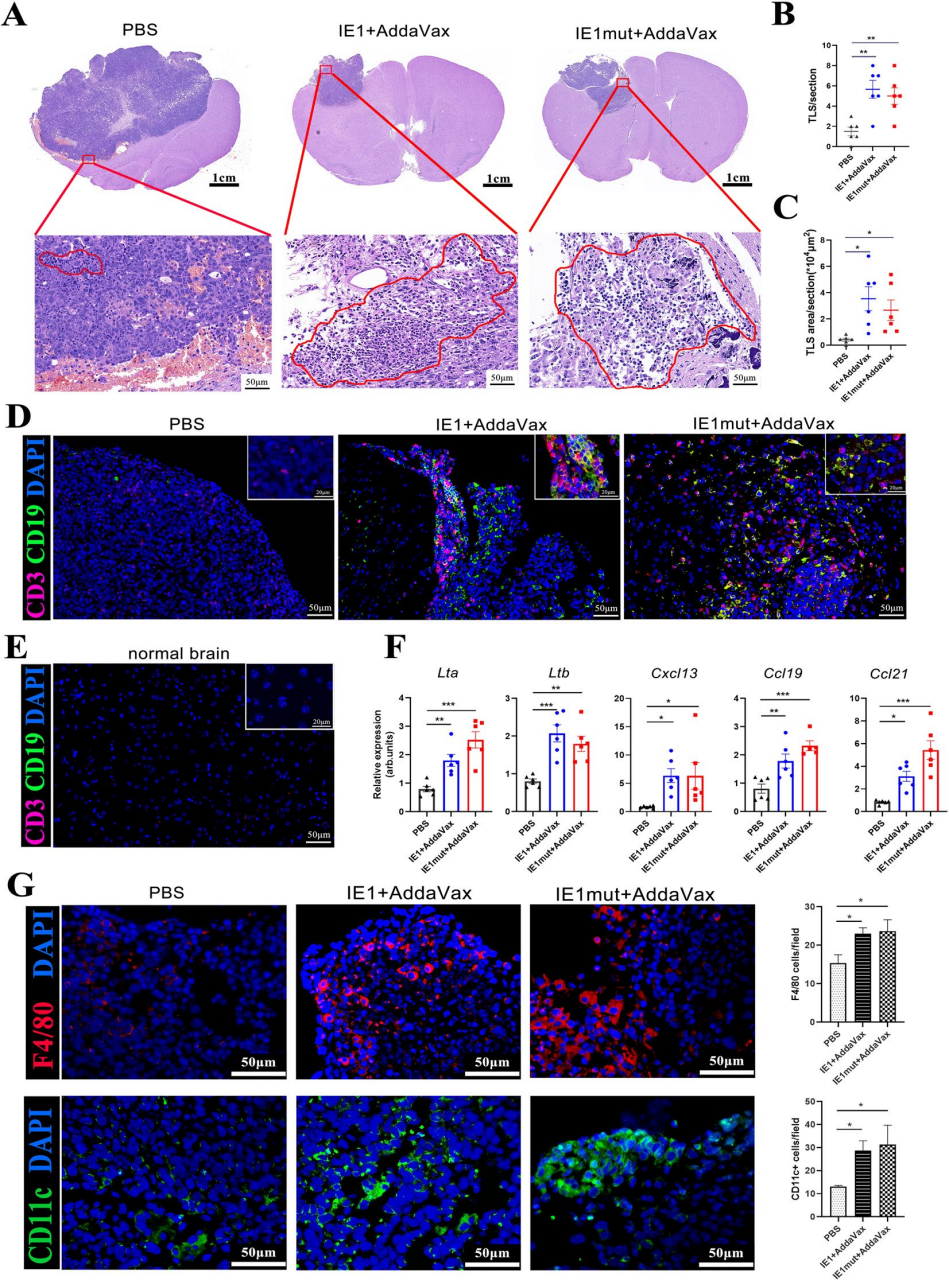
IE1 or IE1mut promotes the formation of TLS in the brain tumor of the GBM model. **A** HE staining of brain tissue sections and the markers of lymphocyte clusters. Scale bar: 50 μm. **D–E** Representative immunofluorescence staining of quantified CD3^+^CD19^+^ cell clusters in the GL261-IE1 model. **B–C** Quantification of the number and areas CD3^+^CD19^+^ clusters brain tumor. *n*=4–6 mice per group. Bars: mean ± SEM. **F** Gene expression of control group and treated group (*n*=6/group) in tumor tissue. arb. units = arbitrary units. **G** Brain tumor sections stained with immunofluorescence for F4/80^+^ macrophages or CD11c^+^ dendritic cells (left). Quantification of F4/80^+^ macrophages or CD11c^+^ dendritic cells (right): red, F4/80; green, CD11c; blue, DAPI. Scale bar: 50μm. Bars: mean ± SEM. The one-way ANOVA was used to analyze statistical differences. *p < 0.05, **p < 0.01, ***p < 0.001

Immunosuppression of the tumor microenvironment is a major obstacle in achieving successful antitumor immunity. To investigate the effect of therapeutic inoculation of IE1 and IE1mut proteins on the tumor microenvironment, these two were intramuscularly injected into orthotopic tumor-bearing C57BL/6 mice with similar tumor sizes along with an equal volume of PBS. Hematoxylin-eosin (H&E) staining was performed on the brain slices of glioma-bearing mice, and clusters of lymphocytes were observed within the tumor (Fig. 3A). Immunofluorescence staining confirmed the presence of prominent B and T cells within these immune cell clusters, leading to TLS (Fig. 3D). TLS is a dense cluster of CD3^+^ T cells and CD19^+^ B cells [11]. In the glioma model with IE1overexpression, the numbers and total surface area of TLS were significantly increased compared with those in the control (PBS) group (Fig. 3B, C). However, no such lymphocyte cluster-like structures were detected in the normal brains tissues (Fig. 3E). Interestingly, we observed that the degree of tumor infiltration was significantly lesser in the treated groups than in the control (PBS) group. To characterize the gene expression signatures associated with TLS [12, 24], we extracted brain tumors from the protein-treated mice and extracted RNA from the collected tissues. Compared with the control (PBS) group, gene lymphotoxin β (*Ltb*), lymphotoxin α (*Lta*), C-X-C-motif chemokine ligand 13 (*Cxcl13*), and C-C-motif chemokine ligand 19 (*Ccl19*), C-C-motif chemokine ligand 21 (*Ccl21*) were highly expressed in the brains of mice in the treated group with a large number and area of TLS (Fig. 3F). Moreover, compared with those of control group, the number of dendritic cells and macrophages infiltrating the brain tumors of mice treated with IE1 or IE1mut exhibited a significant increase (Fig. 3G). In addition, high levels of TNF-α and CD107a expression were found in tumor tissues of the treated group (Supplementary Fig. 2). Overall, IE1 or IE1mut protein treatment enhanced the formation of TLSs, featuring a well-developed B-cell core and T-cell domain.

### IE1 or IE1mut Proteins Trigger the Aggregation and Activation of Peripheral Cytotoxic T Lymphocytes

**Fig. 4:**
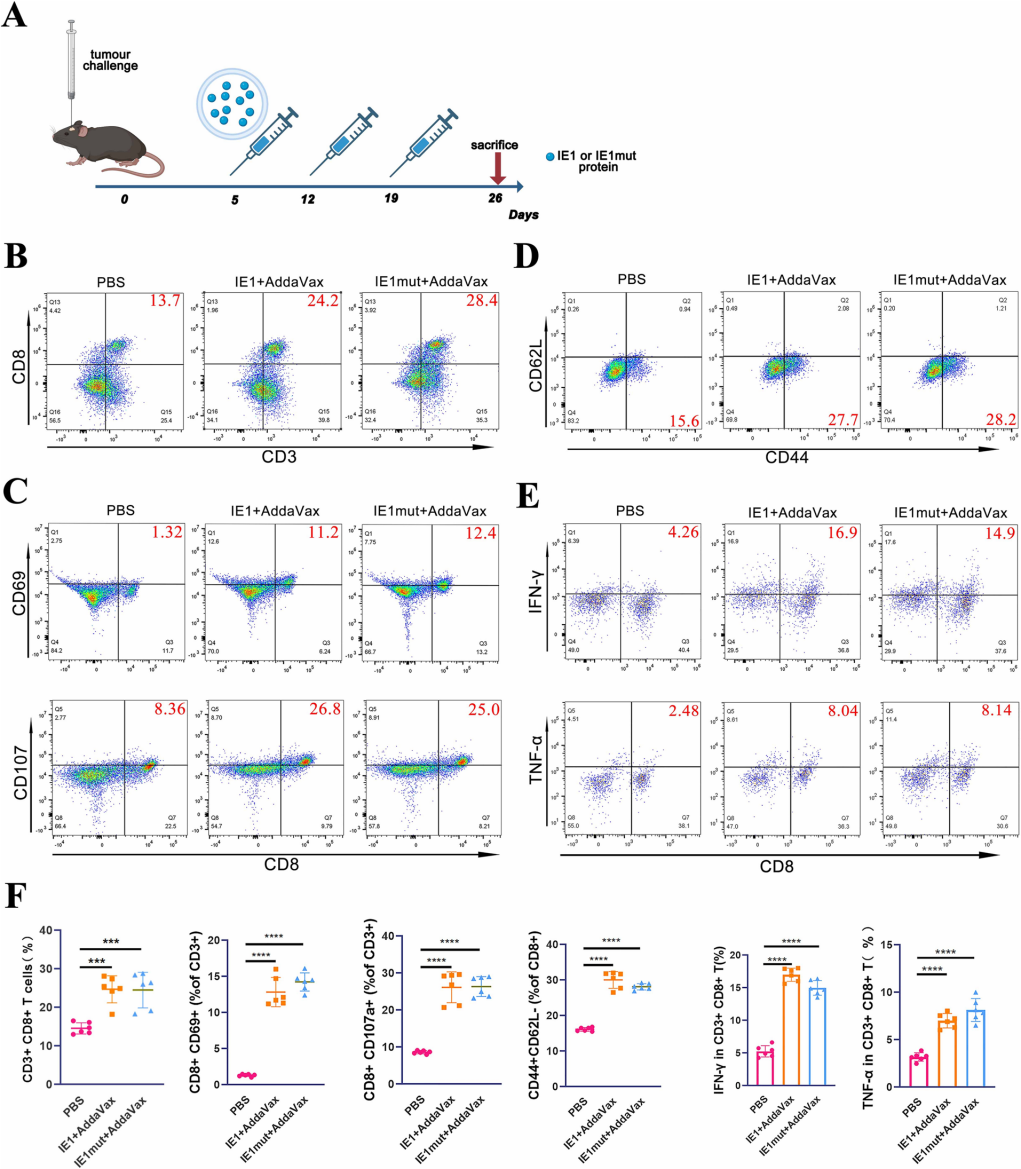
CTL activation after immunization with IE1 or IE1mut. **A** Seven days after 3 times immunization, lymphocytes were isolated to detect T cell activation by flow cytometry. **B–E** Detection of CD8^+^ T cell activation and differentiation. **F** Statistical analysis for the proportion of activated and differentiated CTL cells. *n*=4–6 mice per group. Bars: mean ± SEM. The one-way ANOVA was used to analyze statistical differences. *p < 0.05, **p < 0.01, ***p < 0.001, ****p< 0.0001

To determine whether the IE1 or IE1mut treatment actives the T cells in peripheral the immune organs, the expression of CD8, CD69, CD107a, CD44, CD62L, IFN-γ, and TNF-α were evaluated in the cervical draining lymph nodes and spleen on the 28th day of implantation in tumor-bearing mice (Fig. 4F). To verify the differentiation and function of T cells, the proportion of T cells in CD3^+^ cells was evaluated. The number of tumor-specific CD8^+^ CTLs in the draining lymph nodes was higher than that in the control group (Fig. 4B); moreover, the proportions of CD69^+^CD8^+^ CTLs and CD107a^+^CD8^+^ CTLs with toxic functions were increased (Fig. 4D). Additionally, among the CD8^+^ T cells, the number of CD44^high^CD62L^low^ TEM cells was approximately two times higher than that in the control group (Fig. 4C). Compared with the control group, the secretion of the cytokines IFN-γ and TNF-α was enhanced in CD8^+^ CTL cells (Fig. 4E). Enhanced CTL presence, functional activation, and TEM polarization were observed not only in the deep cervical draining lymph nodes, but also in the spleen (Supplementary Fig. 3). Taken together, these data strongly indicate that IE1/IE1mut treatment induces peripheral CTL activation and differentiation in the glioma model.

### IE1 or IE1mut Proteins Stimulate the Proliferation of Intratumoral T Cells and Lead to Tumor Regression

**Fig. 5:**
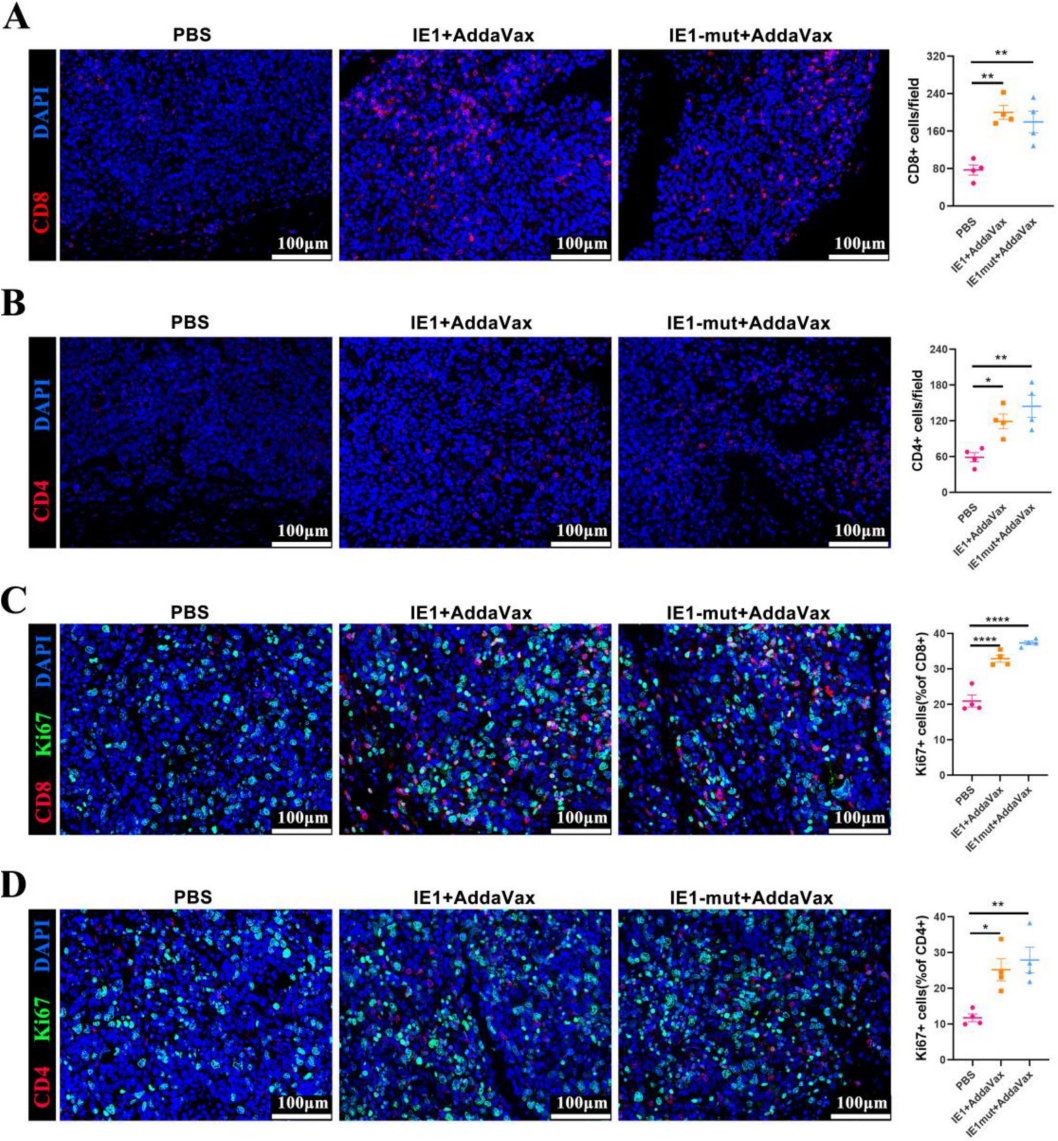
Infiltration and proliferation of CD4^+^ and CD8^+^ cells in brain tumors. **A–B** Immunofluorescence detection of CD4^+^ or CD8^+^ cells in brain tumor sections (left) and quantification of the number CD8^+^ or CD4^+^ cells in per field (right). **C–D** Immunofluorescence detection of CD4^+^ Ki67^+^ or CD8^+^ Ki67^+^ cells in brain tumor sections (left) and the ratio of CD8^+^Ki67^+^ double-positive cells in CD8^+^ cells or the ratio of CD4^+^Ki67^+^ double-positive cells in CD4^+^ cells in each field (right). *n*=4–6 mice per group. Bars: mean ± SEM. The one-way ANOVA was used to analyze statistical differences. *p < 0.05, **p < 0.01, ***p < 0.001, ****p< 0.0001

Immune cell depletion represents is a significant contributor to treatment failure in solid tumors. To investigate the response of T cells during protein therapy, we examined the infiltration and proliferation of T cells in the tumor. Notably, after three protein immunizations, the IE1 and the IE1mut protein treatment groups showed a substantial increase in CD8^+^ T cell infiltration in the tumor compared with the control group (Fig. 5A). In addition, the infiltration of CD4^+^ T cells was also increased in these treatment groups (Fig. 5B). Nevertheless, the number of CD8^+^ T cell infiltrated was higher than that of CD4^+^ T cells infiltrated. Notably, within the CD8^+^ T cell and CD4^+^ T cell populations, there was a significant increase in the proportion of Ki67^+^ T cells was also observed (Fig. 5C, D). In therapeutically vaccinated mice, no evidence of shrinkage in the spleen, deep cervical draining lymph nodes, or immunological organs such as the thymus was observed compared with the control group (Supplementary Fig. 4A–C). Generally, these findings demonstrated that the immune system of the mice receiving immunotherapy was stimulated by IE1/IE1mut treatment, resulting in a long-term antitumor impact.

### IE1 or IE1mut Protein Induces Aggregation and Activation of B Cells

**Fig. 6:**
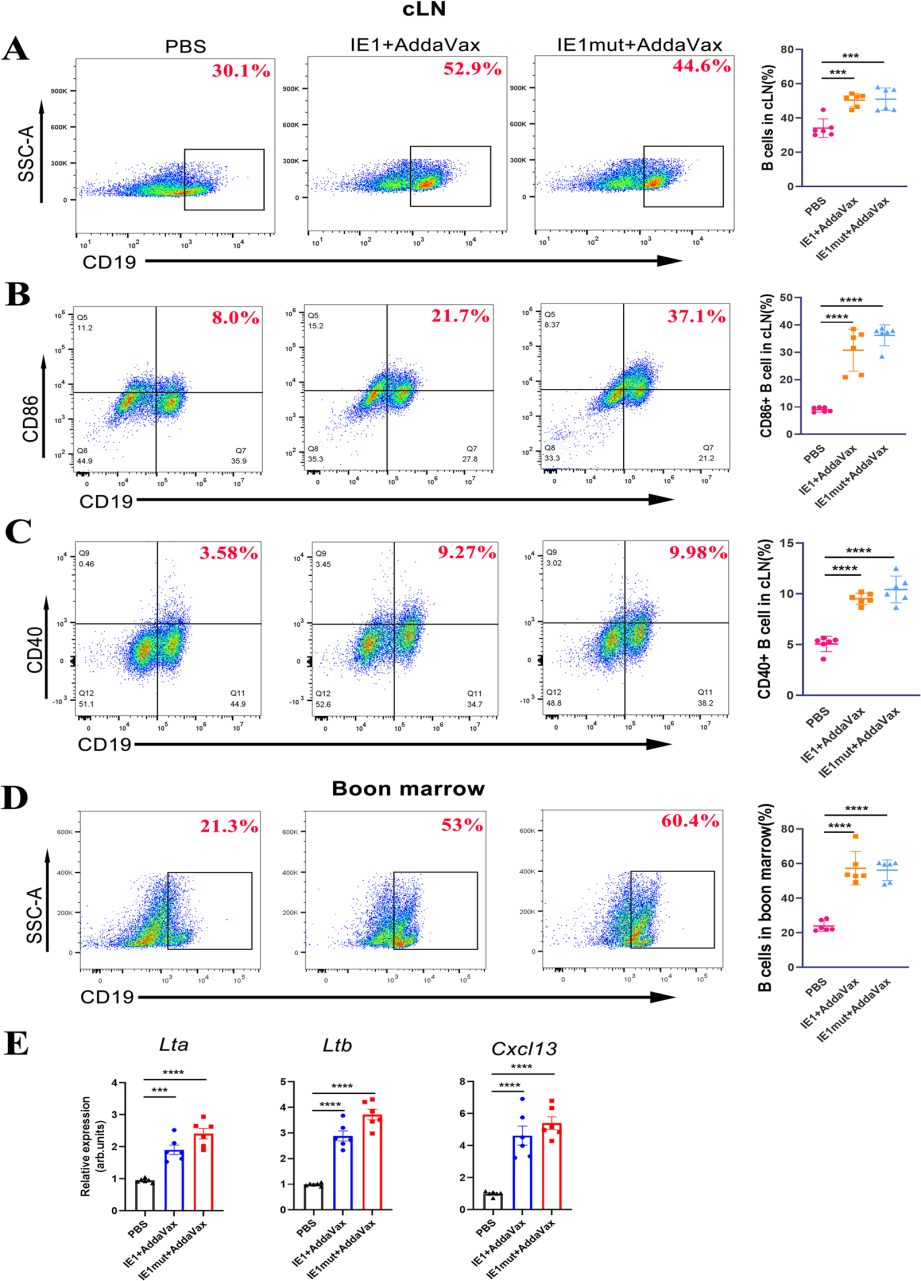
IE1 or IE1mut induces differentiation and function of B cells. **A** Proportion of CD19^+^ cells in cervical draining lymph nodes (left) and quantification of the CD19^+^ cells in cervical draining lymph nodes (right). **B–C** Proportion of CD19^+^CD80^+^ and CD19^+^CD40^+^cells in cervical draining lymph nodes (left) and quantification of proportion of CD19^+^CD80^+^ and CD19^+^CD40^+^cells in cervical draining lymph nodes (right). **D** Proportion of CD19^+^ cells in boon marrow (left) and quantification of proportion of CD19^+^ cells in boon marrow (right). **E** Gene expression of control group and treated group in cervical draining lymph nodes. *n*=4–6 mice per group. arb. units = arbitrary units. Bars: mean ± SEM. The one-way ANOVA was used to analyze statistical differences. *p < 0.05, **p < 0.01, ***p < 0.001, ****p< 0.0001

Prior research has demonstrated that T cell dependent antitumor immune responses play a crucial role in inducing tumor regression [25]. Studies have shown that B cells are required for inducing brain tumor regression [26] and that TLS formation is required in the B cell compartment [27]. Considering the role of B cells in antitumor immunity, we examined the status of B cells in draining cervical lymph nodes by flow cytometry at seventh days after 3 times immunization. Our data indicates that the proportion of B cells in treated groups were higher than that in the control (PBS) group (Fig. 6A). To explore the role of B cells in antitumor immunity, we detected the immune markers on B cells and observed a that the antigen-presenting markers CD86 and CD40 were highly expressed on the surface of B cells (Fig. 6B, C). However, since B cells mature in the bone marrow, we further investigated the B cells in the bone marrow; our results showed that a large number of B cells were present in the bone marrow (Fig. 6D). Simultaneously, we extracted RNA from the cervical lymph nodes to detect the expression of B cell-related genes. The expression of *Ltb*, *Lta*, and *Cxcl13* was significantly increased in the treated group (Fig. 6E). Both the proportion and function of CD19^+^ B cells in the treated groups further validated that the IE1/IE1mut treatment induces B cell aggregation and activation.

## Discussion

Previous evidence has demonstrated that all regions of the HCMV genome are present in a vast majority of GBM samples [28]. The HCMV IE1 protein is highly expressed (76.1%) in glioma tissues [29] and is strongly correlated with the development of aggressive gliomas. In the present study, we developed IE1 and IE1mut vaccine to treat glioma. Our study findings indicated that the IE1mut vaccine had the same immunotherapy effect as IE1 and also enhanced the formation of TLS in the brain and activated the peripheral immune response in the glioma model.

HCMV IE1 has been shown to stimulate robust and specific CD4^+^ and CD8^+^ T cell responses [30]. Nevertheless, IE1 in HCMV could enhance the stability of FEN1 and promote the genomic DNA replication of HCMV, and posttranslational modification of IE1-72 kDa by conjugation to SUMO-1 occurs at a single acceptor site (K450), whereas HCMV of the IE1 mutant reduces the binding between IE1 and FEN1, inhibiting the ubiquitination of IE1and SUMOylation in infected cells, making it less efficient at infecting cells; it also grows more slowly and is produced in lesser amounts in human fibroblasts [22, 31]. In a spontaneous glioma mouse model, Soroceanu et al. [9] demonstrated that the expression of the *IE1* gene (UL123) specifically increased the Sox2 and Nestin levels in IE1-positive tumors as well as upregulated stemness and proliferation markers *in vivo*. Therefore, we suspected that the IE1 protein might be harmful to the body. In the *in vitro* established human and mouse glioma cells overexpressing IE1 or IE1mut, we found that the proliferation and migration ability of IE1mut-overexpressing group was indeed lower than that of IE1-overexpressing group (Supplementary Fig. 1). As a result, we mutated these amino acid positions as described to develop IE1mut based on the IE1 to remove the possible potential harm of the IE1 protein [22, 31]. Delightfully, the serum biochemical results also showed that either IE or IE1mut caused no considerable damage to the body (Fig. 1C), and IE1mut has the same effect as IE1 in terms of immunotherapy. Based on the strict species specificity of HCMV, to simulate HCMV infection of glioma patients and express the IE protein, we constructed a glioma cell line stably expressing the IE1 using lentiviral vectors and successfully established orthotopic glioma models.

Recently, the discovery of TLS in solid tumors was proved has enhanced prognosis of tumor patients [15]. In glioma therapeutic studies, intratumoral generation of TLS was found to facilitate immunotherapy and induce tumor regression [32]. Moreover, the number and area size of TLS were positively correlated with the patients’ survival time. Consistent with their study, we used HE staining to identify the presence of a large number of lymphocyte clusters in the tumor and then characterized the TLS with CD3^+^ and CD19^+^, which was mostly formed in the tumor marginal area in the treated groups. However, no such structures were detected in the other half of the normal brain tissues. In the untreated control group, TLS was also present, but its number and area size were much lower than those of the treated group. In peripheral immune organs, LT-α/β is required for the development and function of stromal cells in the B-cell and T-cell compartments [24, 33]. Cxcl13, also known as B lymphocyte chemokine, selectively induces chemotaxis on B cells and exhibits its function by interacting with the chemokine receptor CXCR5 [34]. Immune and stromal cells can both produce Ccl19 and Ccl21, which may stimulate lymphocytes and aid in the TLS development [35]. In the present study, the expressions of Lta and Ltb were elevated following IE1 or IE1mut vaccination, and the expressions of Cxcl13, Ccl19, and Ccl21 were upregulated in tumors; these findings were consistent with the finding that it promotes T and B cells gathered and facilitates the creation of TLS. According to earlier studies, B cells from the bone marrow are drawn to the brain tumor’s microenvironment where they can gather antigens, transport them to the draining lymph nodes, and present them to T cells. These B cells are essential antigen-presenting cells (APCs) in the immune response [26]. In our study, a large number of B cells were detected in the draining cervical lymph nodes. Nicolas et al. [36] confirmed that B cells play an important role in the pathogenesis of central nervous system autoimmunity independent of their humoral involvement and direct B–T interaction *in vivo*. Moreover, B cells are more efficient APCs when they recognize the same target Ag as the responding T cells. Consistent with our observations of an increased expression of CD40 and CD86 on the surface of B cells in cLNs, it is suggesting that B cells are activated. Moreover, we discovered a high proportion of B cells in the bone marrow. Based on these findings, IE1 or IE1mut treatment induces the recruitment of B cells into the brain tumor microenvironment, serving as essential APCs in the immune response against brain tumors.

In the present study, neither IE1 nor IE1mut protein was found to cause parenchymal organ damage in mice, but they activated the MHC-II lysosomal pathway of antigen-specific CD4 T cells through TH1 response and drove antigens pass through the MHC-I pathway, leading to the activation of cytotoxic CD8 T cells and enhancing antitumor T cell responses [37]. However, immune cell depletion is a major feature of glioma patients with glioma, which is also a major reason for the failure of antitumor immunity. We observed that the thymus, deep cervical draining lymph nodes, spleen, and other immune organs of tumor-bearing animals were all smaller than those of control animals; this finding was consistent with that of previous studies [38]. Conversely, the immune systems of the mice in the treated group did not deteriorate and even grew stronger, suggesting that the IE1 or IE1mut protein stimulates immune cell proliferation and differentiation in immune organs for a considerable amount of time after immunization. However, proliferating CD8^+^ T-cell infiltration is associated with improved survival in patients with glioma [39]. Contrarily, we found a large number of infiltrating CD8^+^ T cells in the brain tumors after immunization with the IE1 or IE1mut protein, and the proportion of proliferating CD8^+^Ki67^+^ T cells increased. CD4^+^ T cells play an indispensable role in antitumor immunity. As helper cells, they can significantly enhance the efficacy of CD8^+^ CTLs in killing tumor cells and promote their differentiation into long-lived memory tumor-specific CD8^+^ T cells [40]. Indeed, we found the presence of higher proliferative CD4^+^Ki67^+^ T cells. It is well known that T-cell activation was essential for the antitumor function. And the CTL mainly through the degranulation or death receptor pathway expresses FasL and secretes TNF specifically to kill tumor cells. Interesting, CD8^+^ cells were successfully activated and secreted to produce more IFN-γ and TNF-α than the control group. Apart from this, we noted that numerous CD8^+^TEMs are present in the peripheral immunological tissue. Altogether, the IE1 or IE1mut protein promoted T cell polarization, T-cell activation, and proliferation, ensuring robust and long-term antitumor activity and assisting in the prevention of recurring cancers. However, it needs to be stressed that due to the strict species specificity of HCMV, there are still certain differences between the overexpression of IE1 in the glioma model we established and the state of HCMV recurrence and expression of IE1 in glioma patients. Therefore, further therapeutic vaccination is needed in clinical patients to observe its clinical treatment effect.

**Fig. 7:**
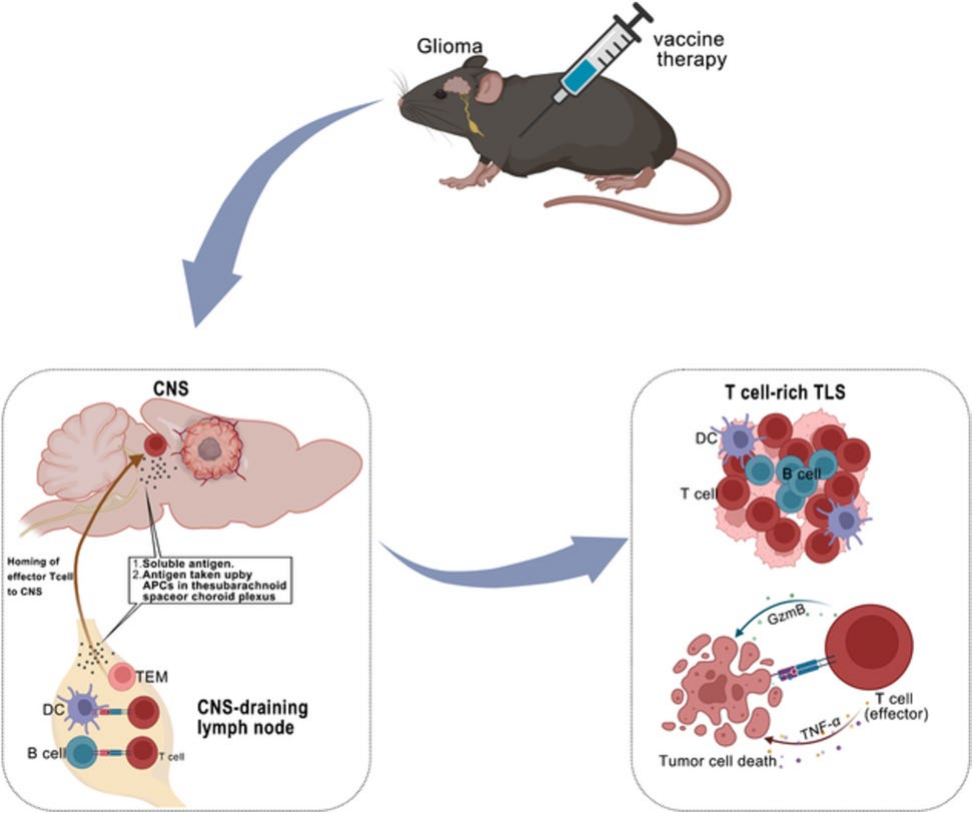
Schematic diagram of IE1/IE1mut activating antitumor immune response to treat glioma

In conclusion, our data suggest that the immunization with a therapeutic glioma vaccine targeting HCMV IE1 is a promising treatment option, and we developed IE1mut that reduces the potential harmful effects of IE1 and exhibits the same immune effects, promotes the formation of TLS and antitumor immune response, continuously kills tumor cells, leads to tumor regression, and extends the survival rate of the glioma model expressing HCMV IE1 (Fig. 7). Our study data provide a new target for the clinical treatment of glioma and offer relevant information for the current research on HCMV in glioma patients.

### Supplementary Information

ESM 1 (PDF 2148 kb)

## Data Availability

The original contributions presented in the study are included in the article/supplementary material. Further inquiries can be directed to the corresponding author.
