# Supplementary material for: HCMV IE1/IE1mut Therapeutic Vaccine Induces Tumor Regression via Intratumoral Tertiary Lymphoid Structure Formation and Peripheral Immunity Activation in Glioblastoma Multiforme
Source: Mol Neurobiol. 2024 Jan 23;61(8):5935–49. doi: 10.1007/s12035-024-03937-8 (PMC11249408; doi:10.1007/s12035-024-03937-8)
Supplement: Supplementary file 1 — (PDF 2148 kb) [file 12035_2024_3937_MOESM1_ESM.pdf]

Supplementary material

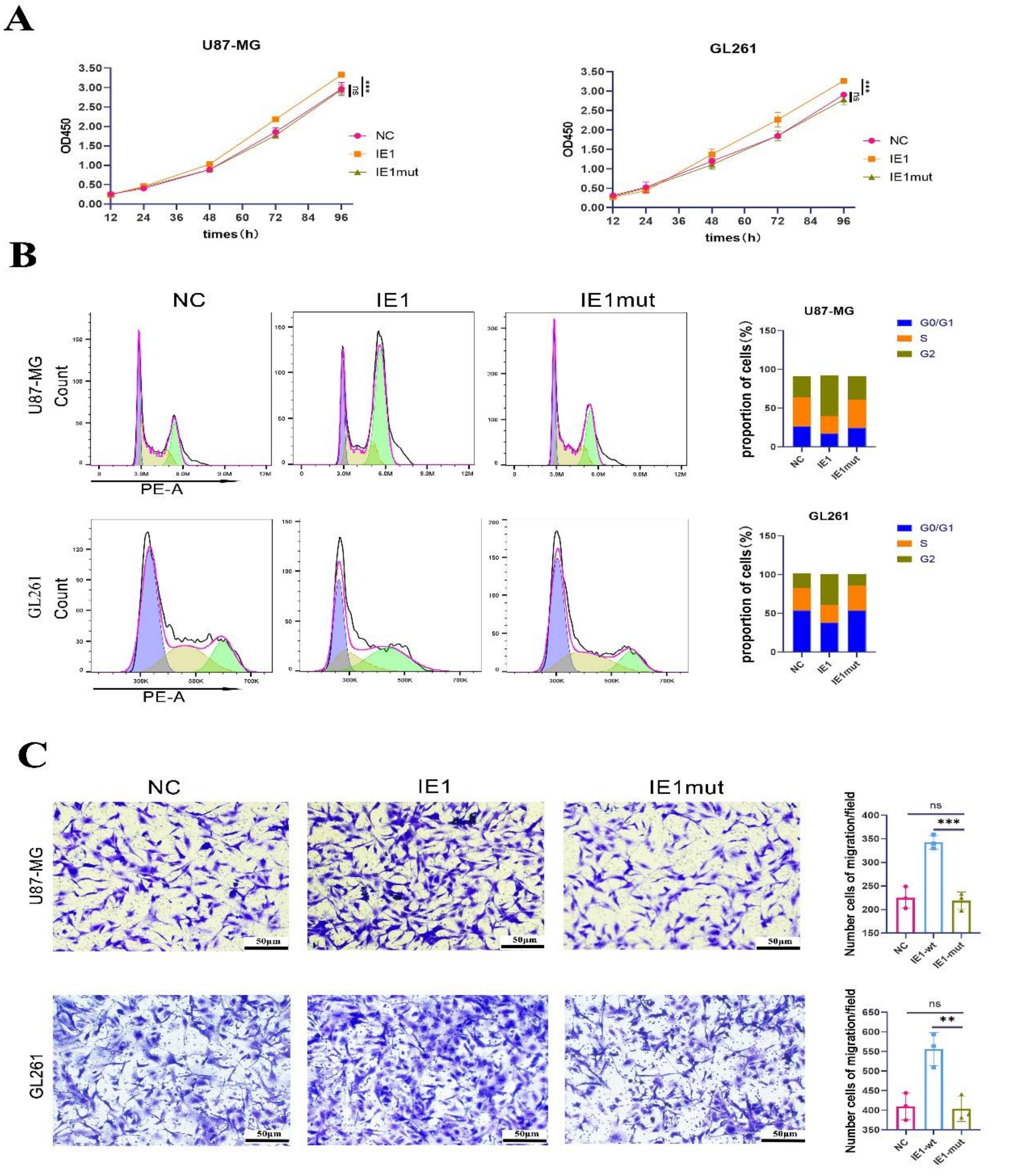

Supplementary Figure 1. IE1 effect cell proliferation and migration *in vitro*. **(A)** Effects of IE1 or IE1mut overexpression on cell proliferation by CCK-8 in U87-MG and GL261 cells. **(B)** Effects of IE1 or IE1mut overexpression on cell cycle by flow cytometry in U87-MG and GL261 cells (left), and proportional distribution of cells in GO, S, and G2/M stages of the cell cycle (right). **(C)**Staining of IE1 or IE1mut overexpression cell migratory capacities by transwell assays in U87-MG and GL261 cells(left), and quantification of migrating cells (right). Bars: mean ± SEM. The one-way ANOVA was used to analyze statistical differences. *$p < 0.05$, **$p < 0.01$, ***$p < 0.001$.

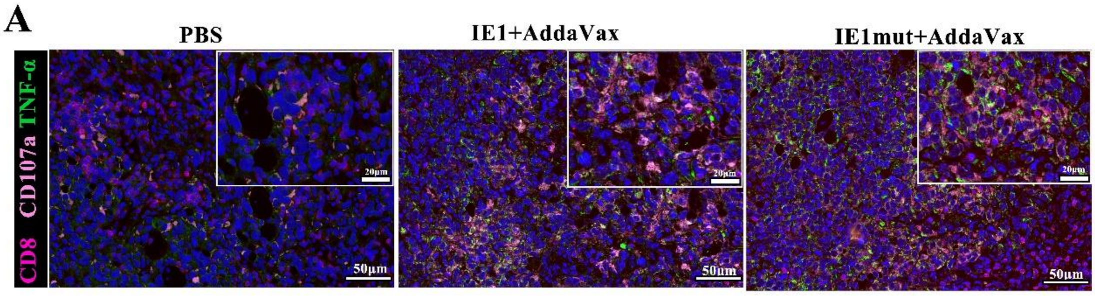

Supplementary Figure 2. CTL kills tumor cells in GBM. **(A)** Representative immunofluorescence staining of CD8, CD107a, and TNF-α in tumor tissue. Red: CD8, pink: CD107a, green: TNF-α, blue: DAPI. Scale bar: 50μm.

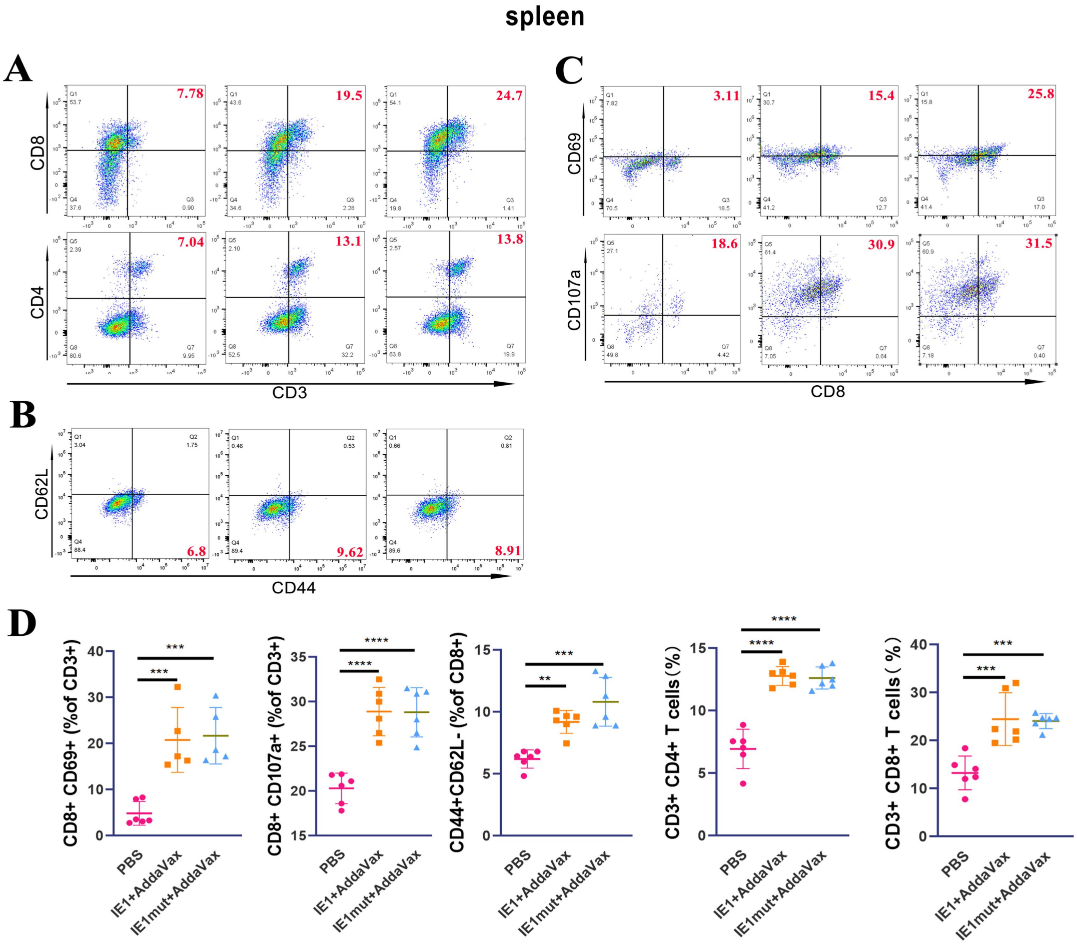

Supplementary Figure 3. Activation of CTLs in the spleen. **(A)** Among CD3^{+} positive cells, the proportion of CD4^{+} and CD8^{+} cells. **(B)** In CTL, the ratio of CD44^{high}CD62L^{low} cells. **c)** Among CD8^{+} positive cells, the ratio of CD69^{+} and CD107a^{+} cells. **(C)** Statistics on the proportion of activated and differentiated CTL cells in spleen. n=6 mice per group. Scale bar: 1cm.Bars: mean ± SEM. The one-way ANOVA was used to analyze statistical differences. *$p < 0.05$, **$p < 0.01$, ***$p < 0.001$, ****$p < 0.0001$.

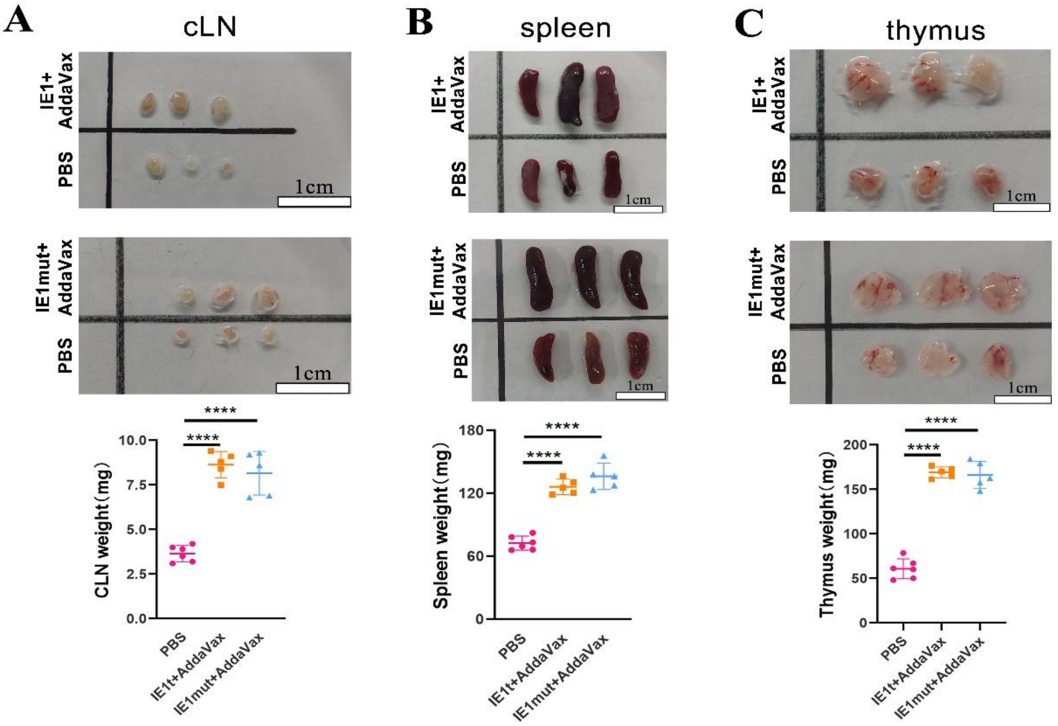

Supplementary Figure 4. Shrunken peripheral immune organs in tumor-bearing mice. **(A)** Comparison of the size and quality of cervical drainage lymph nodes between the treatment group and the control group. **(B)** Comparison of the size and quality of spleen between the treatment group and the control group. **(C)** Comparison of the size and quality of thymus between the treatment group and the control group. n=6 mice per group. Scale bar: 1cm.Bars: mean ± SEM. The one-way ANOVA was used to analyze statistical differences. *$p < 0.05$, **$p < 0.01$, ***$p < 0.001$, ****$p < 0.0001$.

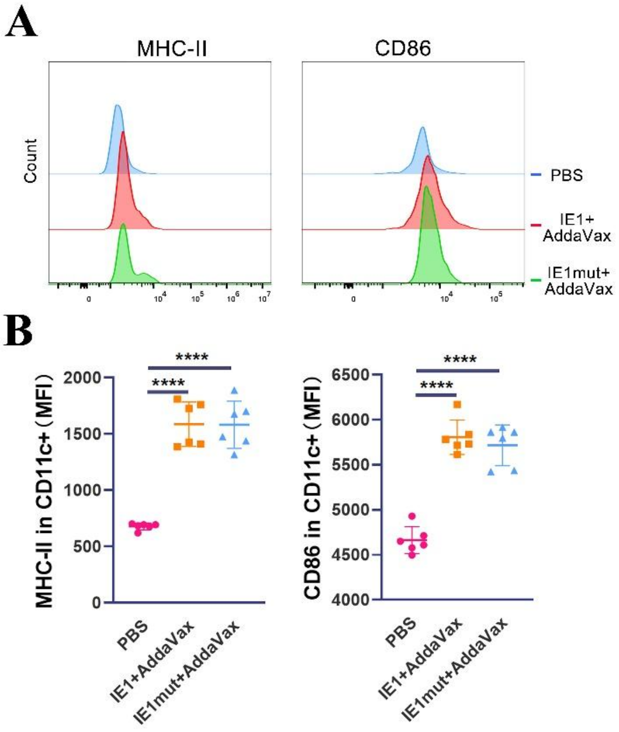

Supplementary Figure 5 Activation of DC in the cLN. **(A)** The histograms of flow cytometric analyses of MHCII and CD86 in CD11c^{+} cells. **(B)** Statistical results of MFI for MHC-II and CD86 in CD11c^{+} DC cells.

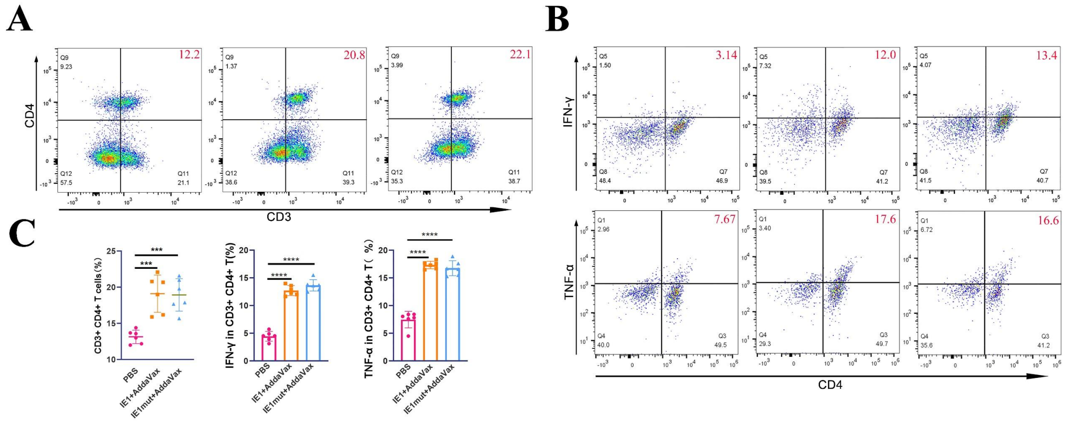

Supplementary Figure 6. IE1 or IE1mut activates CD4^{+} T cells in the cLNs. **(A)** The proportion of CD3^{+}CD4^{+} T cells in cLNs. **(B)** CD4^{+}T activation was assessed by detecting the secretion of IFN-γ, TNF-α. **(C)** Statistics on the proportion of activated and CD4^{+}T cells in spleen. n=6 mice per group. Scale bar: 1cm. Bars: mean ± SEM. The one-way ANOVA was used to analyze statistical differences. *$p < 0.05$, **$p < 0.01$, ***$p < 0.001$, ****$p < 0.0001$.

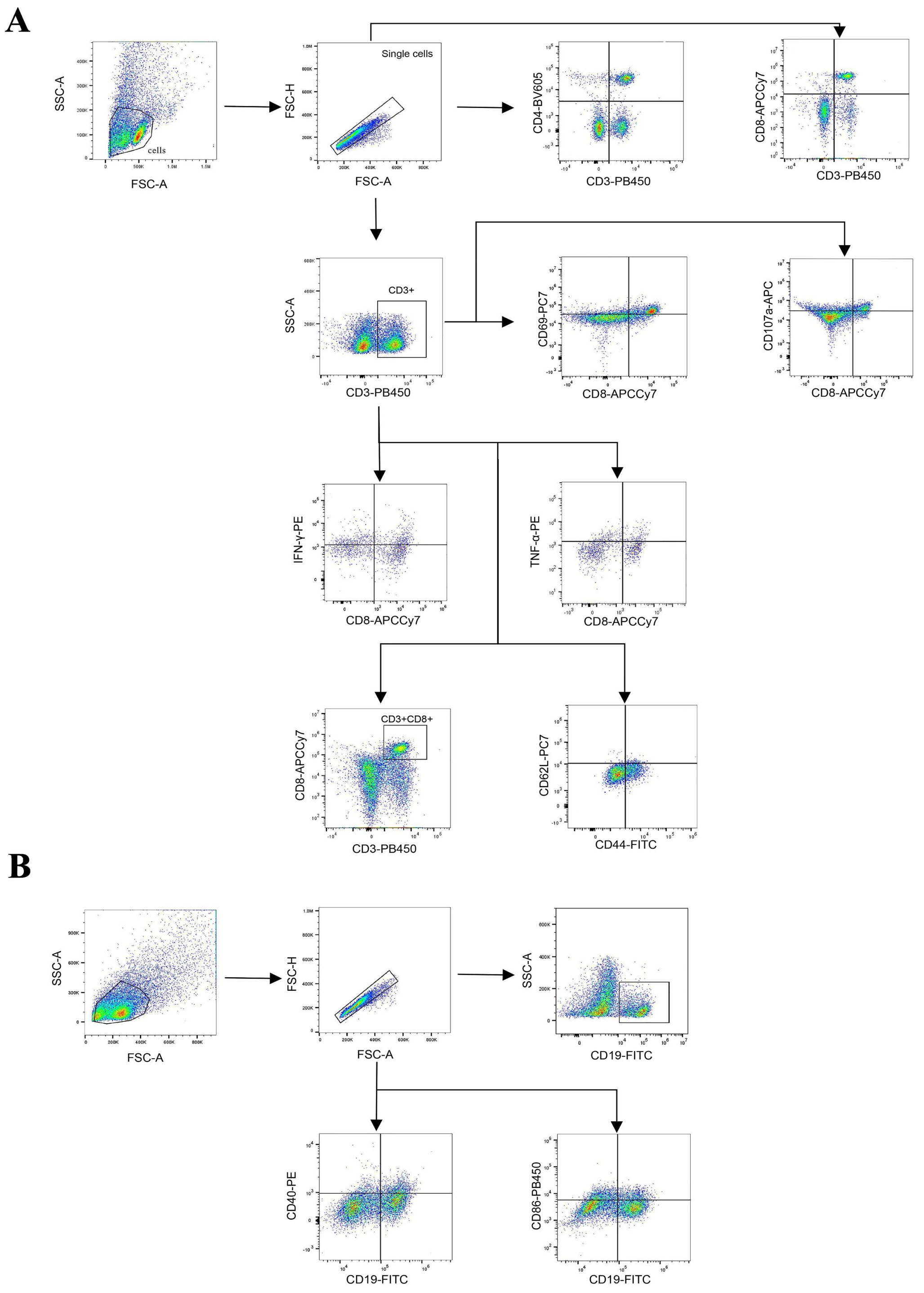

Supplementary Figure 7. Gating strategy used for flow cytometry analysis of Lymphocytes. **(A)** Gating strategy used to identify CD3$^+$CD4$^+$ T cells, CD3$^+$CD8$^+$ T cells, CD69$^+$CD8$^+$ T cells, CD107a$^+$ CD8$^+$ T cells, TNF-α$^+$ CD8 T cells, and IFN-γ$^+$ CD8 T cells in Figure 4 B, C, E. **(A)** Gating strategy used to identify CD44^{high}CD62L^{low} in CD3$^+$CD8$^+$ T cells in Figure 4 D. **(B)** Gating strategy used to identify and CD19$^+$ B cells, CD86$^+$ CD19$^+$ B cells and CD40$^+$ CD19$^+$ B cells in Figure 6.
